# Healthcare Access and County-Level COVID-19 Mortality: Exploring the Impact of Hospital Proximity and Uninsurance Rates

**DOI:** 10.3390/healthcare12242543

**Published:** 2024-12-17

**Authors:** Gabriel A. Benavidez, Anja Zgodic

**Affiliations:** 1 Department of Public Health, Robbins College of Health and Human Sciences, Baylor University, Waco, TX 76798, USA; 2 Department of Epidemiology & Biostatistics, Arnold School of Public Health, University of South Carolina, Columbia, SC 29208, USA

**Keywords:** COVID-19, mortality, healthcare access, health insurance, hospitals

## Abstract

Background/Objectives: Many social and environmental factors contribute to the disproportionate burden of COVID-19 mortality. Access to healthcare services has not been thoroughly examined as a factor contributing to COVID-19 mortality. This study examines distance to ERs and ICUs, uninsurance rates, and county-level COVID-19 mortality rates. Methods: Using data from the American Hospital Association survey, we identified hospitals providing emergency and intensive care services. Hospital locations were geocoded, and straight-line distance was calculated from the population-weighted county centroid. The county proportion of uninsured residents came from the American Community Survey. Generalized linear regression models with a log-link were used to examine study factors and county COVID-19 mortality rates. Results: A total of 2640 (84.0%) U.S. counties or county-equivalents were included in this analysis. The median COVID-19 mortality rate was 240 deaths per 100,000. In adjusted models, increasing distance to ERs (IRR: 0.95; 95% CI: 0.92, 0.98) or ICUs (IRR: 0.61; 95% CI: 0.57, 0.65) was not significantly associated with increased COVID-19 mortality. The proportion of residents (IRR: 3.81; CI: 2.58, 5.62) uninsured was significantly associated with increased COVID-19 mortality rates. Conclusions: Being in close proximity to hospital-based healthcare services may not provide any significant benefit for COVID-19 mortality outcomes, considering that hospitals are largely located in more densely populated areas conducive to COVID-19 spread. Financial barriers may largely contribute to persons avoiding necessary COVID-19 care. To continue to combat COVID-19 and future pandemics, greater attention should be focused on eliminating financial barriers to receiving medically necessary care.

## 1. Introduction

In the United States (U.S.), the burden of the COVID-19 pandemic has disproportionately affected historically marginalized groups, specifically racial and ethnic minoritized people and those of lower socioeconomic status (SES) [1]. The COVID-19 mortality rate for Black, American Indian, and Hispanic people is approximately two times higher than for non-Hispanic White people [2]. Similarly, counties with the lowest socioeconomic status experienced COVID-19 mortality rates two to three times higher than those with the highest socioeconomic status [3]. The combination of race and socioeconomic status has also been shown to play a significant role in the disproportionate mortality burden. Feldman and Basset (2021) showed that if all racial and ethnic minoritized groups had died at the same rate as college-educated White people, there would have been 89% fewer deaths among those aged 25 to 64 in this population [4].

Some evidence suggests that the high prevalence of chronic comorbidities (i.e., diabetes, obesity, hypertension) among racial and ethnic minoritized people and persons of lower SES is one of the primary reasons for disparities in COVID-19 mortality [5]. Lower vaccination rates among this population have also been shown to contribute to higher COVID-19 mortality rates [6]; however, this reasoning fails to acknowledge the structural and environmental inequities that have likely caused the disparities in comorbidities and vaccination uptake. Holden and colleagues highlight the importance of understanding how place-based disparities—where people work, live, and the healthcare resources they can access—are major contributing factors in the observed COVID-19 disparities, especially among historically marginalized groups [7].

A place-based factor that has not been well-studied as a potential contributing factor to COVID-19 mortality is hospital access, specifically proximity to emergency rooms and intensive care units, which are the primary sites for acute and complex care of those with severe COVID-19. Studies have shown that for those at high risk of COVID-19 mortality, the timing of care receipt is crucial to improving the likelihood of survival [8]. Unfortunately, distance to care and affordability can become major barriers for those needing early intervention for COVID-19. To better understand COVID-19 mortality disparities, evaluating how access to care may play a role is critical. Therefore, in this study, we aim to examine the relationship between distance to the nearest hospital-based service (emergency rooms (ERs) and intensive care units (ICUs)) and county-level COVID-19 mortality rates. Additionally, because access to care also encompasses the ability to afford care, we examine the association between county-level uninsurance rates and COVID-19 mortality.

## 2. Materials and Methods

### 2.1. Data Sources

Multiple county-level data sources were used and combined for this analysis. Data on COVID-19 mortality were obtained from the National Center for Health Statistics [9]. We extracted data on COVID-19 mortality from 1 January 2020 to 1 April 2022. Data on the proportion of county residents who were fully vaccinated were obtained from the Centers for Disease Control and Prevention [10]. Data on sociodemographic characteristics were collected from the U.S. Census Bureau’s American Community Survey 5-year (2016–2020) estimates [11]. Hospital-based ER and ICU location data came from the 2019 American Hospital Association (AHA) survey [12], the most robust and comprehensive survey of hospital-based services in the U.S. In addition, missing data from the AHA survey were supplemented with data from the Centers for Medicare and Medicaid Services provider of services files, which contain location data for all hospital facilities that accept Medicaid or Medicare payments [13]. Data on county-level chronic disease prevalence were extracted from the CDC PLACES dataset [14].

### 2.2. Data Preparation

County-level COVID-19 mortality, the proportion of fully vaccinated residents, and sociodemographic characteristics were merged using the county federal information processing standard (FIPS) codes. COVID-19 mortality rates were calculated per 100,000 county residents. Some counties were excluded (n = 503) due to suppressed values for small counts of COVID-19 mortality in accordance with confidentiality requirements from the National Center for Health Statistics. Hospital-based ERs and ICUs were identified by hospitals’ self-reports of providing emergency or intensive care unit services. Hospitals that were flagged for reporting the services of interest were geocoded using ArcGIS’(Redlands, CA, USA) World Geocoding Service. Population-weighted county centroids were created to calculate distances to the nearest ER or ICU. Population-weighted centroids are preferred over geometric centroids as they often can cause bias in distance calculations for sparsely populated areas outside a small number of cities [15]. We then used the closest facility analysis tool in ArcGIS Pro to calculate the distance to the nearest (in miles) hospital-based ER and ICU unit. For additional sensitivity analysis, we also calculated driving time to the nearest hospital, as travel distances in miles and minutes may differ significantly in urban and rural areas. Data from counties in Alaska and Hawaii were excluded from all analyses because the travel mode in these states, especially for hospital-based services, is often by small plane or boat. Previous analyses focused on distances to healthcare services have excluded Alaska and Hawaii for similar reasons [16].

### 2.3. Statistical Analysis

The dependent variable of interest for this analysis was the rate of COVID-19 mortality per 100,000 residents. Three main independent variables were examined individually in this analysis: (1) distance to the nearest ER, (2) distance to the nearest ICU, and (3) proportion of county residents without any form of health insurance. The following county-level covariates were also examined and included in all models to adjust for potential confounding: rural/urban status, the proportion of residents fully vaccinated (2-dose regimen or 1-dose regimen depending on the vaccine received), prevalence of diabetes, prevalence of obesity, prevalence of hypertension, and county median age.

Median values and interquartile ranges are used to describe county characteristics. To model the relationship between county-level COVID-19 mortality and distance to the nearest ER, ICU, and the proportion of uninsured residents, we considered negative binomial, Poisson, and log-link linear models. Based on model diagnostics, including residuals vs. predictions, residual quantile-quantile, scale-location, and leverage point plots, linear regression models with a log-link were deemed most appropriate and were used for all study models. For all models, we used a significance level of α = 0.05, and all confidence intervals are 95% CIs. Bivariate models were used to examine the independent relationship between distance to hospital-based services, the proportion of uninsured residents, and county COVID-19 mortality rates. Multivariable models were then used to adjust for potential confounding variables. Because of the wide variation in county population size across the U.S., all models were weighted for county population size, where the weights were rescaled to have a mean of 1.

### 2.4. Sensitivity Analyses

To assess model fit, all models considered (Poisson, negative binomial, log-link linear) were examined with a random intercept accounting for county clustering within the state and with an Intrinsic Conditional Auto-Regressive (ICAR) state-level random intercept. Both types of random intercepts account for state-to-state variability and the nested structure of counties, the unit of analysis for this study. The ICAR random intercept also takes into account the spatial dependence between states. We used a battery of diagnostic procedures to assess each model of the sensitivity analysis, including hypothesis tests on random intercepts where applicable, residual plots, quantile-quantile plots, and leverage point plots. All sensitivity analyses confirmed that random intercepts did not contribute to the goodness-of-fit of the model and that the linear models with a log-link used in this analysis provided the best fit to the data.

To assess the robustness of our measure of distance, models using distance in miles as the independent variable were rerun to include distance in minutes of travel time as our definition of physical access to hospital-based services. This was examined as we believed there could be large differences in examining travel based on distance or time variables, especially in rural and urban areas. All sensitivity checks confirmed that associations of distance and COVID-19 mortality were not sensitive to how distance was measured (miles or minutes).

## 3. Results

This analysis included 2640 (84.0%) U.S. counties or county-equivalents. The median mortality rate across all included counties was 240 deaths per 100,000 (Table 1). The median distance to the nearest ICU and ER was 19.67 and 9.27 miles, respectively. The median driving time to the nearest ICU and ER was 27.75 and 16.77 min, respectively. The median resident age of the counties included was approximately 41 years. The median prevalence of obesity and diabetes for included counties was 36.20% and 10.50%, respectively. The average proportion of uninsured residents for included counties was 8.40%.

The adjusted models (Table 2) accounted for several county-level covariates to control for potential confounders in the relationship between healthcare access and COVID-19 mortality. Specifically, the models were adjusted for the proportion of residents who were fully vaccinated, median age, median income, prevalence of diabetes, prevalence of obesity, and rural or urban status of the county.

In adjusted models, an increased distance to the nearest ICU was associated with lower COVID-19 mortality overall (IRR: 0.61, 95% CI: 0.57–0.65, *p* < 0.0001). This inverse association persisted when stratified by rural and urban counties, but the effect was stronger in urban counties (IRR: 0.60, 95% CI: 0.55–0.65, *p* < 0.0001) compared to rural counties (IRR: 0.85, 95% CI: 0.78–0.92, *p* < 0.0001). Similarly, greater distance to the nearest ER was associated with lower COVID-19 mortality overall (IRR: 0.95, 95% CI: 0.92–0.98, *p* < 0.0001), with a more pronounced effect in urban counties (IRR: 0.85, 95% CI: 0.82–0.88, *p* < 0.0001) than in rural counties (IRR: 0.98, 95% CI: 0.92–0.99, *p* < 0.0001).

The proportion of uninsured residents was positively associated with COVID-19 mortality at the overall level (IRR: 3.81, 95% CI: 2.58–5.62, *p* < 0.0001). This effect also varied between rural and urban counties, with a stronger association observed in urban counties (IRR: 4.03, 95% CI: 3.02–5.43, *p* < 0.0001) compared to rural counties (IRR: 2.87, 95% CI: 1.98–3.59, *p* < 0.0001).

In sensitivity analyses examining driving time, ICU driving time was associated with lower COVID-19 mortality overall (IRR: 0.98, 95% CI: 0.98–0.99, *p* < 0.0001), with no significant association in rural counties (IRR: 1.06, 95% CI: 0.98–1.15, *p* = 0.102) but a significant effect in urban counties (IRR: 0.88, 95% CI: 0.81–0.94, *p* < 0.0001). For ER driving time, the overall IRR was 0.98 (95% CI: 0.98–0.99, *p* < 0.0001), with rural counties showing no significant association (IRR: 1.04, 95% CI: 0.92–1.17, *p* = 0.109) and urban counties showing a significant association (IRR: 0.91, 95% CI: 0.82–0.98, *p* < 0.0001).

## 4. Discussion

This analysis examined the association between county-level COVID-19 mortality and healthcare access, defined as physical proximity and the ability to pay using health insurance as a proxy. While many studies have already highlighted consistent and strong associations of common (e.g., income, employment, educational status) social determinants of health and COVID-19 outcomes, this study is one of the first to examine how physical access to healthcare may impact COVID-19 mortality. Notably, this analysis suggests that longer travel distances to the nearest location for both ICU and ER services are associated with significant decreases in county COVID-19 mortality rates. This was true overall and when stratified by rural and urban counties. This finding was replicated in sensitivity analyses when travel time was included in the models rather than travel distance since travel time and travel distance can vary widely. However, there was no observed association between travel time to ICUs or ERs and COVID-19 mortality in rural counties. We did observe that COVID-19 mortality increased as the proportion of county residents without any form of health insurance increased, and this was found in our overall analysis and when stratified by rural and urban status.

Our findings on the negative association between travel distance and COVID-19 mortality appear counterintuitive, as a significant portion of the literature in this area suggests positive health outcomes from convenient access to healthcare services [17,18]. However, in the context of COVID-19, closer proximity to ICUs and ERs might suggest a more suitable environment for COVID-19 transmission and eventual mortality. For example, whether a hospital is non-profit or for-profit, a major factor in determining hospital location is access to a sizable customer base to generate sustainable revenue [19]. This often results in hospitals being located in city centers where population density is relatively higher than in surrounding suburban or metropolitan areas. For instance, in a 2012 study, Hanlon and colleagues showed that as population density increased in geographic areas, there was a significant increase in the availability of maternal and child health service providers [20]. Studies from the U.S. and worldwide confirm that population density significantly drives COVID-19 cases and mortality [21]. Therefore, we hypothesize that the current results may partly be capturing this effect, as living further away from hospital locations likely resulted in less opportunity for COVID-19 transmission and mortality. As approximately 91–95% of households in the U.S. own or have access to a vehicle [22], travel distance and time may not be as vital a measure in the context of receiving COVID-19 care. However, these findings are not uncommon. In a systematic literature review examining the relationship between travel time and distance on various health outcomes, Kelly and colleagues concluded mixed findings [23]. Nearly a fourth of the 108 studies reviewed found a null or inverse effect between travel distance/time and health outcomes [23]. Additionally, another possible explanation of our findings is that in urban and more densely populated counties, hospital systems were severely more overwhelmed with COVID-19 patients and had higher occupancy rates throughout the pandemic compared to rural counties [24]. Studies examined the role of hospital capacity and found that in-patient mortality was associated with hospitals with increased occupancy [25].

Our findings, which show the positive association between the proportion of uninsured residents and COVID-19 mortality, highlight the importance of considering multiple aspects of access. The availability of services may be less important than the ability or perceived ability to afford services when needed. Eberth and colleagues noted this point in a study examining access to multiple hospital-based services among minority populations [16]. Contrary to their initial hypothesis, they found that predominately Black and Hispanic urban neighborhoods were significantly closer in distance to hospital-based healthcare services than White neighborhoods [16], even though traditionally, these populations have had worse health outcomes. A 2002 landmark report from the Institute of Medicine (IOM) was one of the first to provide a comprehensive review of the literature on the impact of healthcare coverage on various health outcomes [26]. The IOM report found consistent evidence that lack of health insurance leads to poorer overall health, shorter lives, and lower quality of care when received [26]. Since the IOM report, studies using more sophisticated causal inference methods have continued to conclude that healthcare coverage is directly linked to improved health outcomes and all-cause mortality [27]. In the context of COVID-19, earlier receipt of care for COVID-19 demonstrated a greater likelihood of survival among patients admitted to the hospital [28]. Previous studies have demonstrated that patients who are concerned about their ability to afford care are three times more likely to avoid or delay receiving medical care [29]. Further supporting our findings, an analysis by Campbell et al. (2022) [30] directly estimated the effect of health insurance coverage on COVID-19 mortality. They estimated that of the 225,000 COVID-19-related deaths occurring between 1 January 2020 and 13 February 2021, approximately 60,000 could have been avoided if patients had adequate health insurance coverage.

The rural–urban distinction in our results provides additional context for the relationship between healthcare access and COVID-19 mortality. We observed a stronger negative association between proximity to healthcare facilities and COVID-19 mortality in urban counties compared to rural ones. This difference may further reflect the impact of population density on COVID-19 transmission, as urban areas generally have a higher concentration of people and a correspondingly greater likelihood of viral spread, even with better access to hospital services. Conversely, rural counties showed a weaker negative association, which might indicate that distance is a less relevant factor for COVID-19 mortality where population density is lower.

While the United States is no longer experiencing significant surges in COVID-19 hospitalizations and mortality due to a combination of population vaccination campaigns and increased seroprevalence of infection-induced SARS-CoV-2 antibodies, these findings demonstrate the importance of examining “access” as a holistic, multidimensional construct that involves multiple domains [31]. Our finding that increased distance to the nearest ICU or ER is not associated with worse COVID-19 outcomes demonstrates that in the context of COVID-19, potential measures of travel burdens may not be the most relevant factor in determining whether individuals are able to seek medical care. However, one’s ability or perceived ability to afford care may be a more accurate conceptualization of access during acute public health emergencies or future pandemics. Policies like the Families First Coronavirus Response Act of 2020, which prohibited the termination of enrollees from Medicaid during the COVID-19 pandemic, likely played a major role in reducing the burden of COVID-19 throughout the United States [32]. Pandemic preparedness plans for future public health emergencies should identify specific health policy components that temporarily expand Medicaid coverage to ensure financial barriers are not prohibitive to seeking care.

A major strength of this study is that it is one of the first to examine the impact of multiple dimensions of healthcare access on COVID-19 outcomes. To our knowledge, no study has examined how distance to hospital-based ER and ICU services may impact COVID-19 mortality. Additionally, this study used both travel distance and time to confirm the robustness of our findings. However, this study is not without limitations. First, this study relies on aggregated county-level data, and findings should not be extrapolated at the individual level (e.g., ecological fallacy) to suggest that travel time or distance to care does not serve as a barrier against positive health outcomes. Additionally, this study only examined the distance to hospital-based ER and ICU units, and other types of COVID-19 care, such as testing or vaccination sites, may have been more important in contributing to county-level COVID-19 mortality. Other hospital-related factors, such as the number of staff available, bed capacity, or other resources, were not considered in this analysis. It is possible that underreporting of COVID-19 deaths may also skew our results, as places with greater distances to care, especially those in rural areas, have been shown to have a greater number of excess deaths that were not attributed to COVID-19. Future studies, perhaps using electronic medical records, could examine the role that travel distance to care may have played in COVID-19 mortality. This would help inform future pandemic preparedness plans.

## 5. Conclusions

This study highlights the importance of reducing financial barriers to healthcare, as higher uninsurance rates were linked to increased COVID-19 mortality in both rural and urban areas. Policy efforts should focus on expanding insurance coverage, especially in regions with high uninsurance. Additionally, expanding community-based healthcare services in rural areas could minimize risks associated with traveling to high-density centers. Tailored pandemic preparedness strategies that address both financial and physical access to healthcare are essential to reducing disparities in future public health crises.

## Figures and Tables

**Table 1:** Characteristics of the included counties.

Variable (n = 2640)	Median (IQR)
Non-Hispanic Black Residents	2.20 (0.60, 9.90)
Non-Hispanic White Residents	83.20 (64.40, 92,00)
Hispanic Residents	4.40 (2.30, 10.00)
American Indian Residents	0.20 (0.10, 0.60)
Asian Residents	0.60 (0.30, 1.30)
Other Residents	0.10 (0.00, 0.20)
Fully Vaccinated	49.90 (42.60, 58.10)
Unemployed	2.80 (2.20, 3.60)
Proportion Uninsured	8.40 (5.80, 11.90)
Diabetes Prevalence	10.50 (9.10, 12.20)
Obesity Prevalence	36.20 (33.50, 38.40)
COVID-19 Death Rate	240.99 (151.64, 361.47)
Median Age	41.40 (38.40, 44.60)
Median Income ($)	52,700 (45563, 61339)
Nearest ICU (miles)	19.67 (4.87, 32.75)
Nearest ICU (minutes)	27.75 (10.95, 42.09)
Nearest ER (miles)	9.27 (3.77, 25.15)
Nearest ER (minutes)	16.77 (8.67, 34.41)

**Table 2:** Unadjusted and adjusted incidence rate ratios.

**Regression Analysis**	**Overall ^a^**		**Rural**		**Urban**	
	**IRR [95% CI]**	***p*-Value**	**IRR [95% CI]**	***p*-Value**	**IRR [95% CI]**	***p*-Value**
Nearest ICU (miles)	0.61 [0.57 0.65]	<0.0001	0.85 [0.78, 0.92]	<0.0001	0.60 [0.55, 0.65]	<0.0001
Nearest ER (miles)	0.95 [0.92, 0.98]	<0.0001	0.98 [0.92, 0.99]	<0.0001	0.85 [0.82, 0.88]	<0.0001
Proportion of Uninsured	3.81 [2.58, 5.62]	<0.0001	2.87 [1.98, 3.59]	<0.0001	4.03 [3.02, 5.43]	<0.0001
**Sensitivity Analysis**	**Overall**		**Rural**		**Urban**	
	**IRR [95% CI]**	***p*-Value**	**IRR [95% CI]**	***p*-Value**	**IRR [95% CI]**	***p*-Value**
Nearest ICU (min driving)	0.98 [0.98, 0.99]	<0.0001	1.06 [0.98, 1.15]	0.102	0.88 [0.81, 0.94]	<0.0001
Nearest ER (min driving)	0.98 [0.98, 0.99]	<0.0001	1.04 [0.92, 1.17]	0.109	0.91 [0.82, 0.98]	<0.0001

^a^ Adjusted models include the following county-level covariates as potential confounders: proportion of residents fully vaccinated, median age, median income, the prevalence of diabetes, and the prevalence of obesity.

## Data Availability

The publicly available data used in this study will be made available upon reasonable request to the corresponding author. Data that are not publicly available (e.g., American Hospital Association data) cannot be shared due to restrictions from the proprietary nature of the data.
